# Neural management plus advice to stay active on clinical measures and sciatic neurodynamic for patients with chronic sciatica: Study protocol for a controlled randomised clinical trial

**DOI:** 10.1371/journal.pone.0263152

**Published:** 2022-02-04

**Authors:** Leticia Amaral Corrêa, Juliana Valentim Bittencourt, Maria Alice Mainenti Pagnez, Stephanie Mathieson, Bruno Tirotti Saragiotto, Gustavo Felicio Telles, Ney Meziat-Filho, Leandro Alberto Calazans Nogueira

**Affiliations:** 1 Rehabilitation Science Postgraduation Program, Augusto Motta University Centre (UNISUAM), Rio de Janeiro, Brazil; 2 Institute for Musculoskeletal Health, Sydney School of Public Health, Faculty of Medicine and Health, The University of Sydney, Sydney, Australia; 3 Universidade da Cidade de São Paulo (UNICID), São Paulo, Brazil; 4 Physiotherapy Department, Federal Institute of Rio de Janeiro (IFRJ), Rio de Janeiro, Brazil; Prince Sattam Bin Abdulaziz University, College of Applied Medical Sciences, SAUDI ARABIA

## Abstract

Advice to stay active is the primary management strategy for sciatica. Other conservative treatments such as neural management techniques may also contribute to sciatica recovery, but currently, the effects have not been robustly assessed. Thus, the aim of this study is to compare the effects of adding neural management to advice to stay active versus advice to stay active alone in improving pain intensity and functional limitation. Secondarily, to compare the effects of the experimental intervention in the sciatic neurodynamic, pain modulation, and psychosocial factors. A parallel-group, controlled, examiner-blinded superiority clinical trial randomised at a 1:1 allocation will be conducted in 210 participants with chronic sciatica. Patients will be recruited from outpatient physiotherapy clinics and community advertisements. The experimental group will receive neural mobilisation techniques and soft tissue mobilisation techniques for 30 minutes per session, 10 weekly sessions, plus advice to stay active on their activities of daily living, information on physical activity, imaging tests, and sciatica for 5 biweekly sessions lasting 25–30 minutes. The control group will receive advice to stay active only. The re-evaluation will be performed out after 5 weeks, 10 weeks, and 26 weeks after randomisation and primary endpoints will be pain intensity and functional limitation at 10 weeks. Secondary outcomes will include neuropathic symptoms, sciatic neurodynamic, pain modulation, and psychosocial factors. Adverse events and patient satisfaction will be assessed. Ethical approval has been granted from an Institutional Human Research Ethics Committee.

**Trial registration**: Trial was prospectively registered in the Brazilian Registry of Clinical Trials (number: RBR-3db643c).

## Introduction

Low back pain (LBP) is the leading cause of years lived with disability worldwide [1]. Patients with chronic LBP usually present co-occurring pain [2]. In primary care, the prevalence of patients with back-related leg pain varied from 48% to 74% depending on the used description [3]. Sciatica is also known as lumbosacral radiculopathy that presents as a severe form of LBP characterised by radiating leg pain and is considered chronic when symptoms last more than 12 weeks [4]. Patients with sciatica have a worse prognosis, higher incidence of disability compensation, greater pain intensity and functional limitations compared to patients with localised LBP [5–8]. The success of the treatment is dependent on various identified prognostic factors such as sociodemographic (e.g. male), clinical (e.g. longer duration of LBP, comorbidities), lifestyle (e.g. smoker), and psychosocial factors (e.g. kinesiophobia) [9]. These prognostic factors are important considerations to identify appropriate treatment and management of patients with sciatica.

The treatment of sciatica is challenging. Although treatment should be patient-centred and aligned with clinical practice guidelines, local pathways can make it difficult for patients to achieve proactive capability to manage their sciatica [10]. Clinical practice guidelines and systematic reviews recommend several conservative treatments as the initial treatment for patients with sciatica [11–13]. Referring acute sciatica patients to physical therapy improve clinical outcomes [14]. Conservative treatment strategies aim to reduce pain intensity and maintain function. There is evidence that active conservative treatment is effective in patients with severe sciatica [12, 15], and advice to stay active continues to be a first-line recommendation for the management of sciatica in most international clinical practice guidelines [13, 16]. Other conservative strategies, such as exercise, has been found not to be superior to advice to stay active in reducing pain intensity and disability of people with sciatica [17]. However, neurodynamic treatment has shown a significant reduction of leg pain intensity and improved function after four weeks of intervention in patients with chronic nerve-related leg pain, although there was no differences between interventions on the primary outcome after two weeks [18].

There is growing evidence that neural management may facilitate recovery in patients with sciatica. Neural management aims to restore peripheral nervous system homeostasis through nerve and surrounding tissue mobilisation [19, 20]. Neural management may assist in the restoration of the neurodynamic of the sciatic nerve. Compression of the sciatic nerve may be associated with changes in nerve function [4]. Besides, the improvement of clinical symptoms in sciatica patients is related to the normalisation of nerve mobility [21]. A decrease in intraneural oedema and improvement in temporal summation and median nerve latency were presented after neural management for different conditions (e.g. nerve-related neck and arm pain, tarsal tunnel syndrome, and plantar heel pain) [22]. Neural involvement may be investigated by ultrasound imaging. The use of ultrasound images to assess the properties of nerves is becoming increasingly popular [23]. Ultrasound images quantify the nerve neurodynamic, including excursion and the cross-sectional area of the nerve [24–26]. Thus, ultrasound images represent a resource able to assess the effect of neural management treatment on nerve structure.

The benefits of neural management as a treatment strategy in patients with chronic sciatica is currently limited by low-quality evidence. Neural management has shown positive results for improving pain and function in patients with LBP [22, 27, 28] and to be superior to other treatments of exercise, conventional physiotherapy, traction or manual therapy for patients with chronic nerve-related musculoskeletal pain such as sciatica, chronic radicular LBP, nerve-related neck and arm pain, and carpal tunnel syndrome [29]. Previous studies using neurodynamic treatment presented methodological limitations as high population heterogeneity [27] and small sample size [18, 28]. Despite being a frequently used strategy, there is a lack of good methodological studies to demonstrate the clinical effects and benefits of neural management for patients with chronic sciatica. Therefore, we propose a randomised controlled clinical trial to analyse the effects of adding neural management to advice to stay active versus advice to stay active alone in medium-term (10 weeks) improvement of pain and function limitation for patients with chronic sciatica. Secondarily, the study will compare the effects of adding neural management to advice to stay active versus advice to stay active alone in the neuropathic symptoms, sciatic neurodynamic, pain modulation, and psychosocial factors.

## Materials and methods

### Study design

A two-armed, randomised, controlled, examiner-blinded, superiority clinical trial. The study will be conducted according to the requirements Consolidated Standards of Reporting Trials (CONSORT) [30] and Standard Protocol Items: Recommendations for Interventional Trials (SPIRIT) (S1 File) [31].

### Registry

This trial was approved by the Research Ethics Committee of Augusto Motta University Centre (number: 40500720.8.0000.5235), in accordance with the Helsinki Declaration for research in humans and was prospectively registered in the Brazilian Registry of Clinical Trials (number: RBR-3db643c).

### Setting

The trial will be conducted in the Augusto Motta University Centre outpatient physiotherapy, located in Rio de Janeiro, Brazil.

### Recruitment

Participants will be recruited when they seek the outpatient physiotherapy due to chronic sciatica and by community adverts in the local population. Potential participants could be screened by phone to determine eligibility before the assessment session. All patients who met the eligibility criteria will sign the informed consent form at the recruitment process.

### Eligibility criteria

Potential participants will be assessed by a physiotherapist (M.A.P.) to confirm the diagnosis of sciatica. We will include adults (18–65 years old) with chronic sciatica with moderate or severe leg pain intensity (greater than 3 on the Numerical Pain Rating Scale) [32, 33]. Sciatica will be defined as pain radiating unilaterally to the lower limb below the knee, with symptoms of paraesthesia along the path of the sciatic nerve, involving the dermatomes of L4 to S1, in addition to possible neurological symptoms persistent or recurrent for more than three months [4, 7, 34]. The inclusion criteria will also include the presence of higher pain intensity in the lower limb compared to the lumbar region, being symptomatic at the time of assessment, and clinical evaluation to confirm the diagnosis of chronic sciatica. The clinical diagnosis will be confirmed if the participant has two or more positive findings based on 5 items: positive neural tension of the sciatic nerve, neurologic deficit, pain below the knee, pain intensity higher in the lower limb compared to the lumbar region, and subjective sensorial changes [33].

The participants will be excluded if they are already in treatment due to sciatica or planning to undergo any other procedures for sciatica treatment in the study period, present known or signs of serious spinal pathology (i.e., cauda equine syndrome, trauma, fractures, or cancer); history of abdominal, lumbar, or lower limb surgery in the past year; rheumatologic disease in the acute inflammatory phase; if they are receiving disability compensation due to LBP; pregnant or puerperal women; self-reported psychiatric diagnosis; and history of cancer or cancer-related pain.

### Recruitment and enrolment strategies

Participants will be recruited from outpatient physiotherapy clinics. We will use different recruitment methods, including physiotherapist referrals who assist the patients in outpatient physiotherapy, community adverts in the local population, and internet adverts. All participants will receive a flier explanatory about the advantage, rights, duties and responsibilities of the participants included in the research. Participants will be recruit continuously until the desired sample size is reached.

### Randomisation, allocation, and blinding procedures

After the initial assessment, participants will be randomised to one of two groups; neural management plus advice versus advice alone (1:1 allocation). The allocation sequence will be prepared a priori using the "Research Randomizer", an online random number generator available at https://www.randomizer.org/. The randomisation of the participants will be performed using randomly permuted blocks of 4 and 6.

An independent examiner not involved in the study recruitment, assessment, or data analysis will allocate participants to the randomised group to ensure secret allocation from participants. Concealed allocation will be granted using numbered, sequentially ordered, sealed opaque envelopes with an index card written in which intervention group the participant will be enrolled. The envelopes will be numbered consecutively. The same examiner will open the sealed envelopes after the informed consent form has been completed, and the participant carries out the initial assessment. The participants will receive a unique study enrolment number and be referred to the physiotherapist responsible for the intervention group in which the participant was allocated.

Subsequently, the interventions will be performed by the physiotherapist’s authors (J.V.B. and G.F.T.) at the outpatient physiotherapy. The physiotherapists will be blinded to the initial assessment. The physiotherapist (M.A.P.) responsible for assessing pre-treatment, during, and post-treatment will be unaware of randomisation and be blinded to treatment allocation. Statistical analysis will also be conducted blinded to group allocation. Due to the nature of the interventions, physiotherapists and participants will not be blind to treatment allocation. To guarantee that the treatment expectation is evenly balanced between the groups, the participants will not know the study hypothesis. The unblinding of the study will occur in case any serious adverse event occurs or once the completion of the research and the data analysed and interpreted.

### Intervention

The experimental group will receive advice to stay active associated with neural management, and the control group will receive advice to stay active alone. The experimental protocol will be performed by two trained physiotherapists, blinded to the initial assessment (J.V.B. and G.F.T.) and will be conducted according to the requirements Template for Intervention Description and Replication (TIDieR) [35].

#### Advice to stay active

Experimental and control participants will receive the same advice to stay active protocol. In the first session, all participants will receive a blank booklet of activities developed by the researchers, in order to register their activities of daily living performed during the week (e.g., eating, cooking, bathing, brushing teeth, shaving, changing clothes, housework, financial management, proper medication, time away from home, and social interaction activities). This booklet of activities should be presented to the physiotherapist in the next session. Also, both experimental and control group will receive the session at Augusto Motta University Centre (UNISUAM) outpatient physiotherapy. Participants will be submitted to the same protocol and there will be no modification of the protocol during the study.

The intervention will be performed by a physiotherapist (J.V.B.) with 3 years of clinical experience. All participants will receive five sessions of advice to stay active in their daily living activities, information on physical activity, imaging tests, and sciatica. These sessions will be performed in person and individually, lasting 25–30 minutes [36], and there will be a gap of two week between the sessions. Advice to stay active will focus on two themes: potential harmful effects of pain and beneficial effects of stay active on pain [18]. In the harmful effects of pain, we will discuss avoidance of daily-life activities [18, 37, 38], prolonged rest [18], sedentary lifestyle and imaging [11, 39]. In the beneficial effects of stay active on pain, we will discuss the benefits of staying active [17, 39–43], how to avoid bed rest and the positives aspects of returning to work as soon as possible [39, 40, 44], practising physical activity [39, 40, 45], understanding sciatica, pain tolerance and favourable prognosis [17, 40]. In the first four sessions of advice to stay active, one discussion point of harmful effects and one discussion point of beneficial effects will be addressed in each session. Participants will receive a summary of the content covered in all previous sessions at the last session.

#### Neural management

Neural management will be delivered individually by a physiotherapist (G.F.T.) with 9 years of clinical experience. Participants will receive neural mobilisation techniques and soft tissue mobilisation techniques for 30 minutes per session, 10 weekly sessions, considering the participant’s availability. All participants in neural management will be treated by the same physiotherapist, and the techniques will be performed at the symptomatic limb. The treatment sessions will be conducted at UNISUAM outpatient physiotherapy.

In each neural management session, participants will receive the following protocol in the affected lower limb, based on a previous study [28]:

- Myofascial release techniques in the piriformis and biceps femoral muscles in the prone position, performed manually by the therapist using the thumb to sliding in the transverse direction of the piriformis muscle for 15 repetitions, and a longitudinal direction of the biceps femoris muscle for 5 repetitions. When locating any tissue barrier (myofascial trigger point), sustained pressure in the region will be applied for 90 seconds.
- Soft tissue techniques including cross-fibre friction technique used to reduce the entrapment of the sciatic nerve along the nerve pathway. Therefore, a friction technique will be applied in the craniocaudal direction on the sacroiliac joint bilaterally for 15 repetitions with the patient in the prone position. The friction technique will be applied with manual contact directly on the ligaments of the sacroiliac joint.
- Hip joint mobilisation techniques will be performed in supine position for 1-minute each technique. The therapist will be at the contralateral side of the affected limb, and the patient will be positioned with a hip flexion around 90 degrees and maximum knee flexion. The asymptomatic limb will be extended. The anteroposterior technique will be performed by mobilising the hip joint with the therapist’s hand located in the knee and performing the posterior glide in the hip joint. The therapist will also perform an internal rotation hip mobilisation, positioned at the same side of the affected limb, and with both hands located in the middle area of the femur.
- Neural mobilisation will be performed after the initial techniques to improve the excursion of the sciatic nerve. Sliding techniques will be performed as neural mobilisation manoeuvres of the sciatic nerve with hip flexion and extension associated with ankle plantar flexion and dorsiflexion. The therapist will perform 3 sets of 30 oscillations for 1 minute, with the patient in supine position and knees extended. Patient will rest for 1 minute among the sets. The range of motion should not cause pain or paraesthesia symptoms, and the range will gradually increase according to the patient’s response to the application of the technique.

### Criteria for discontinuing or modifying allocated interventions

The allocated intervention could be modified or discontinued once a participant requests explicitly or refuse to continue the treatment or follow-up assessment, and the reason will be fully reported. The study will be discontinued in case of serious adverse events (any significant disability, hospitalisation, life-threatening, and death) occur that make continuing the study harmful for the participants regardless if related to the intervention (or control) or not. Ancillary and post-trial care (e.g., provision and/or cover for additional health care of immediate adverse events related to trial procedures) will be provided for participants who suffer sustained harm as a result of their involvement in this trial at no costs.

### Outcomes measures

The primary outcomes will be leg pain intensity and functional limitation. Secondary outcomes will include neuropathic symptoms, sciatic neurodynamic, pain modulation, and psychosocial factors (detailed in S2 File). The follow-ups will be performed in 5 weeks, 10 weeks, and 26 weeks after randomisation. The follow-up assessment at 10 weeks after randomisation will be considered the primary time-point. In addition, adverse events and patient satisfaction will also be assessed.

#### Primary outcome

*Pain intensity*. Pain intensity will be measured using the Numeric Pain Rating Scale (NPRS) from 0 (no pain) to 10 (worst pain possible). Participants will be asked to rate their leg pain and low back pain intensity at the evaluation moment. The NPRS is a common scale in pain studies, including those with sciatica and has good reproducibility levels [46]. The minimally clinically important difference considered in the current study will be an effect size of 0.2. The duration of pain will be recorded in months.

*Functional limitation*. Functional limitation will be investigated using the Patient-Specific Functional Scale (PSFS). The PSFS is a self-reported measure widely used to assess limited functional activities in patients with musculoskeletal disorders, presenting excellent reliability [47]. Participants will be asked to identify up to three important activities they are unable to perform or are restricted as a result of their pain. Each activity will be classified on an 11-point Likert scale regarding the current level of difficulty, which 0 means “unable to perform activity” and 10 “able to perform activity at the level as before injury”, and the mean of the three activities will be calculated. PSFS has ease applicability and can be used clinically as an outcome measure [48, 49].

#### Secondary outcomes

*Neurological assessment*. The neuropathic symptoms will be assessed by a clinical examination for neurological assessment and the painDETECT questionnaire. The clinical examination for neurological assessment will include muscular function and sensory abnormalities evaluation [50–53].

*Sciatic neurodynamic*. The sciatic neurodynamic will be assessed by the range of motion of the hip during the straight leg raise test, the cross-sectional area of ​​the sciatic nerve, and the echogenicity ratio [54–62].

*Conditioned pain modulation*. Cold pressor test (CPT) will assess the conditioned pain modulation (CPM). The CPT is a psychophysical test used to assess the CPM, where the cold water (between 1°C and 4°C) is the pain conditioning stimulus, and pressure pain threshold (PPT) is the test stimulus. The CPT is an appropriate method to assess the descending nociceptive inhibitory system [63, 64].

*Psychosocial factors*. Brief Psychological Screening Questions (BPSQ) will be included to assess the psychosocial factors. The BPSQ is a self-reported questionnaire that assesses the influence of psychosocial factors on an individual’s health. Anxiety symptoms, social isolation, catastrophisation, depression symptoms, kinesiophobia and perceived stress are the domains assessed [65, 66]. In addition, self-efficacy will be evaluated by the isolated item “I am confident I can cope with my condition” [67].

### Adherence to interventions, adverse events, and assessment of patient satisfaction

Adherence to home treatment will be assessed through the activity’s agenda, in which participants must register the activities that were performed at home in the days between the sessions, in order to remain active. High adherence will be defined as ≥ 70% attendance at sessions [68].

Serious adverse events and adverse events that occurred during the intervention will be assessed by a self-reported questionnaire developed by the researchers to register the symptom and/or adverse event, with duration and intensity details. Serious adverse events will include any significant disability, hospitalisation, life-threatening, and death, and will be reported immediately to the researchers and ethics committee.

Patient satisfaction assessment will be performed through MedRisk, a questionnaire capable of measuring the level of satisfaction of the patient who will be receiving interventions through physiotherapists. MedRisk consists of 20 items, 10 items related to the therapist-patient interaction, 2 items that are considered global and 8 items that evaluate other domains. Participants will score patient satisfaction using a Likert scale ranging from 1 (strongly disagree) to 5 (strongly agree). The higher the score, the higher the satisfaction [69].

### Data collection and management

Patient’s characteristics at baseline assessment will be collected immediately before randomisation. All the data collected, and outcomes will be obtained using printed questionnaires. The original data will be scanned as image files by a research assistant and shared with a second research assistant. Data integrity will be audited by regularly scrutinising data for omissions and errors by double entered with automated checks in Microsoft Excel spreadsheet (Microsoft Corporation) performed by L.A.C. remotely. Discrepancies will be explored and resolved by checking the original data.

Participants will be identified by an individual trial number to ensure confidentiality, and confidentiality regarding the data collected in all stages will be guaranteed by stored on password-protected servers. The paper-form data will be stored in locked filing cabinets at the Postgraduate Program in Rehabilitation Sciences. Data will only be accessible to the research team. All statistical analyses will be performed using the individual number of each participant and the statistician will be blinded to the group. The results will be presented by group data and any individual data will be spread to ensure confidentiality is preserved.

#### Baseline assessment

Participants will be referred for baseline assessment consisting of the demographic (age, sex, weight, height, house income, marital state, profession, and education level), clinical (comorbidities, duration of pain, location of pain on the body map, average pain intensity, pain area, and medication), and lifestyle (physical activity and quality of sleep).

Physical activity will be evaluated using the short International Physical Activity Questionnaire (IPAQ) and categorised as low (<600 MET x min/week or <150 minutes per week of moderate intensity physical activity), moderate (600–3000 MET x min/week or 150–750 minutes per week of moderate intensity physical activity), or high (> 3000 MET x min/week or > 750 minutes per week of moderate intensity physical activity) [70, 71].

Sleep quality will be evaluated using the question of sleep quality assessment of the Pittsburgh Sleep Quality Index (PSQI) [72]. Each participant will answer, “During the past week, how would you rate your sleep quality overall?” and rate their self-perception of sleep quality as very good, fairly good, fairly bad, or very bad. In addition, the participant will answer two questions about how pain has interfered with sleep, classifying it in a dichotomous scale (yes or no) if "Are you awake in the middle of the night or early in the morning because of your pain?" and "Have you had trouble falling asleep because of your pain?”.

**Fig 1:**
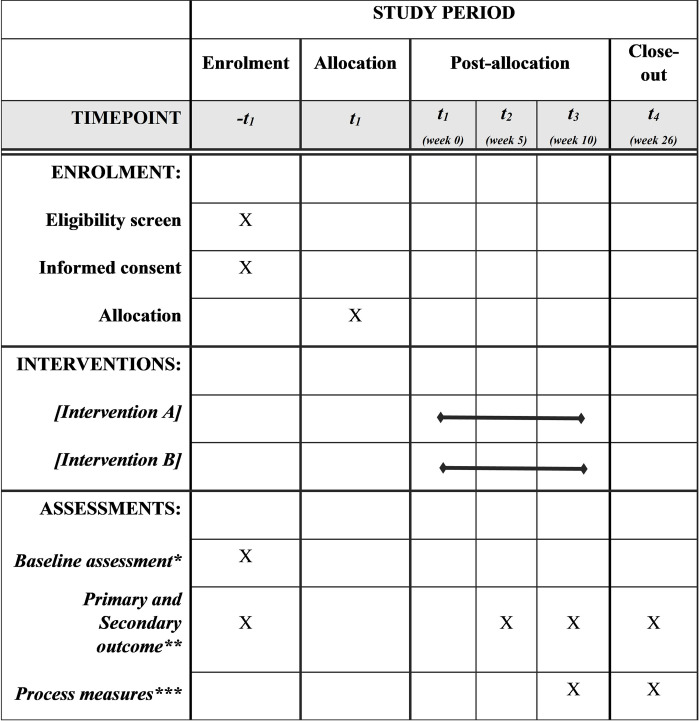
Schedule of enrolment, interventions, and assessments. *Sociodemographic, clinical, and lifestyle characteristics. **Primary outcome: Pain intensity and functional limitation. Secondary outcome: Neuropathic-like symptoms, sciatic neurodynamic, conditioned pain modulation, and psychosocial factors. ***Adherence assessment, serious adverse events and adverse events, and assessment of patient satisfaction.

Data collection will be performed by using a standard questionnaire. The completion of all questionnaires will be supervised by an examiner for clarification in case of uncertainties and will last approximately 30 minutes per participant. The schedule will be performed as presented in Fig 1.

### Statistical analysis

#### Sample size calculation

The sample size calculation was performed a priori in the G*Power software version 3.1 (Heinrich-Heine-Universität, Düsseldorf, Germany) to determine a sufficient sample size. An estimated small effect size of 0.2 [73] in a repeated-measures analysis of variance (ANOVA) was considered to detect the between-groups difference for leg pain intensity (NPRS) and functional limitation (PSFS), with a statistical power of 90% and an alpha of 0.05 (5%), including 2 groups and 3 evaluations (initial, 5 weeks and 10 weeks) and a possible post-randomisation exclusion of 15% [74]. Based on the mentioned assumptions, the estimated sample size was 105 participants per group. A total of 210 participants will be included in the present study.

#### Data analysis

All data will be stored in a Microsoft Excel spreadsheet (Microsoft Corporation) and made available for statistical analyses. Data analysis will be performed by intention-to-treat using multiple imputation technique for missing data analysis and conducted by an independent and blinded examiner. The reasons for missing data will be reported. The demographic and clinical variables of the study population will be presented as mean and standard deviation for continuous variables. Categorical variables will be presented as absolute values and percentage. For continuous variables, the normal distribution of the study outcomes will be verified by the Shapiro-Wilk test. A significance level of less than 5% (p < 0.05) will be considered for all analyses. The statistical analysis will be performed using JASP version 0.10.2.0, and graph analysis will be performed using GraphPad Prism software (GraphPad Software version 8.00 for MacBook, San Diego, CA, USA).

#### Primary analysis

The between-group differences for the primary outcomes considering the change in pain intensity and functionality from baseline to each 5, 10- and 26-weeks after randomisation will be calculated using mixed linear models using the interaction terms of treatment group versus time. The pain intensity and functionality will be investigated in separate models.

#### Secondary analysis

The between-group differences for the secondary outcome considering the change in neuropathic symptoms, sciatic neurodynamic, conditioned pain modulation, and psychosocial factors from baseline to each 5, 10- and 26-weeks after randomisation will be calculated using mixed linear models using the interaction terms of treatment group versus time.

#### Tertiary analysis

The patients’ adherence, serious adverse events or adverse events, and patient satisfaction will be described and presented as mean and standard deviation for continuous variables and as absolute values and percentage for categorical variables.

We will also carry out the mediation analysis to understand whether sociodemographic factor, pain characteristics, and psychosocial factors affect the primary outcomes. The mediator’s analysis will be performed by simple mediation analysis, considering one variable per model, and we will use data collected at baseline. Models will be designed separately for pain intensity and functional limitation, considering the treatment group as independent variable (X), pain intensity and functional limitation as dependent variable (Y), and sociodemographic factor, pain characteristics, and psychosocial factors as mediators (M).

#### Subgroup analyses

No subgroup analysis is planned for this study.

#### Sensitivity analysis

A sensitivity analysis will be performed, including different imputation techniques for missing data to assess the robustness of the primary analysis and results for a per-protocol analysis to assess the efficacy of the protocol.

### Plans for communicating important protocol amendments to relevant parties

Important protocol modifications such as changes to eligibility criteria, outcomes, or analyses will be notified to relevant parties (e.g., Research Ethics Committee, researchers, participants, and journal of publication). Also, participants will be asked to give feedback on any change in protocol. Participants’ feedback regarding the study participation satisfaction will be collected by MedRisk questionnaire at study closure.

## Supporting information

S1 File CONSORT, SPIRIT, and TIDieR checklists. (DOCX)

S2 File Detailed secondary outcomes. (DOCX)

S3 File (PDF)

S4 File (PDF)
