# Supplementary material for: Neural management plus advice to stay active on clinical measures and sciatic neurodynamic for patients with chronic sciatica: Study protocol for a controlled randomised clinical trial
Source: PLoS One. 2022 Feb 4;17(2):e0263152. doi: 10.1371/journal.pone.0263152 (PMC8815873; doi:10.1371/journal.pone.0263152)
Supplement: S2 File — (DOCX) [file pone.0263152.s002.docx]

**Detailed Secondary outcomes**

*Neurological assessment*. The neuropathic symptoms will be assessed by a clinical examination for neurological assessment and the painDETECT questionnaire. The clinical examination for neurological assessment will include muscular function and sensory abnormalities evaluation. Muscle weakness in L5/S1 myotomes will be investigated by “walking on the heels” and “walking on the toes” tasks;(1) bilateral leg perimetry will be measured in the middle region of the leg (15 cm below the lower edge of the patella). The sensitive neurological examination will be performed bilaterally, in the supine position and eyes closed. The vibratory, painful, pressure, and thermal sensitivity examination will be performed. Vibratory sensitivity will be tested using a tuning fork (model C128 Hz) on the bony prominence of the hallux. Painful pinprick sensitivity will be assessed by a stick stimulus in L4, L5, and S1 dermatomes with a needle present in the reflex hammer (Buck model). Pressure sensitivity will be assessed using Semmes-Weinstein monofilaments (SORRI-BAURU^®^, São Paulo, Brazil) of 2g and 10g. Thermal sensitivity will be assessed by two test tubes filled with water (hot - 37ºC and cold - 22ºC) on the participant's lower limb and held for up to three seconds.(2) Grading these sensorial exams will be performed subjectively by the participant in relation to the corresponding contralateral side and classified as: no sensation, decreased sensation, normal, or increased sensation. The painDETECT is a self-administered questionnaire validated for a large number of neuropathic pain conditions that encompass the intensity of pain, pain course pattern, areas of pain and the presence of radiating pain, and sensory descriptor items of pain. A final score between -1 to 38 can be achieved, and for a scores ≤ 12 a neuropathic component is unlikely, whereas, in the ≥ 19 scores, a neuropathic component is probable.(3,4)

*Sciatic neurodynamic.* The sciatic neurodynamic will be assessed by the range of motion of the hip during the straight leg raise test, the cross-sectional area of ​​the sciatic nerve, and the echogenicity ratio. The range of motion of the hip during the straight leg raise test will be assessed using a digital clinometer positioned in the anterior region of the tibia, 5 cm distally from the anterior tibial tuberosity. The clinometer will be positioned so that the examiner who will perform the straight leg elevation test will not see the test result and will remain blind to the measurement. Clinometer application (version 2.8, Peter Breitling, Plaincode) will measure hip movement during the straight leg raise test. The Clinometer application has good intra-tester reliability and moderate to good validity.(5,6) The hip flexion angle will be measured in two moments bilaterally. Participants will be instructed to indicate the first sensation in the posterior thigh, leg or lumbar region (A1) and point of symptoms tolerance (A2). The participant's symptom to be considered may be the sensation of stretching, burning, pricking, or other sensation. This research methodology has been used in several previous studies.(7–10)

Cross-sectional area of ​​the sciatic nerve and echogenicity ratio will be measured using ultrasound (Mobissom, portable MDuo model, São Paulo - Brazil). The images will be taken by a physiotherapist (M.A.P) with 10 years of experience with musculoskeletal ultrasound exams, using the B-mode with a convex transducer at a 10 MHz frequency, with a tracking depth of 60mm and a gain of 30dB. The Mobissom application for iPad^®^  (iPad Air, iOS 12.4.5, Apple) will be used to view, record, and store images. Images will be backed up regularly to ensure no loss of data. The ultrasound transducer will be placed between 5 and 7 cm above the popliteal, transversely to the sciatic nerve, and two copies of each position will be recorded. The first copy will have a digital marker to assist in identifying of the location of the nerve, and a second image will be saved as a clean copy without a digital marker. The procedure will be performed bilaterally and recorded first with the participant in the left lateral position in order to blind the examiner to the symptomatic limb and visualise the sciatic nerve of the thigh that will be above, with the lower limbs flexed at 60° for the hip and 90° for the knee(11) measured by a goniometer. The first image will be recorded with the patient with the knee flexed, the second image will be recorded with the patient with the knee in extension and passive tibiotarsal plantar flexion, and the third image will be recorded with the patient with the knee in extension and passive tibiotarsal dorsal flexion.(12) A portable wooden platform will be placed between the legs, with the top leg resting on the platform. The examiner will be positioned at the rear, with the hand and arm resting on the platform.

The processing of the images will be performed by the ImageJ software (version 1.43, National Institutes of Health, Bethesda, Maryland, USA), which analyses different image formats, calculates the area, and offers pixel statistics, in addition to calibration in centimetres or millimetres. For the assessment of the echogenicity, the images registered will be converted to 8-bit. Each pixel is assigned a grey value ranging from 0 (black) to 255 (white). We will use the histogram-derived performed in the ImageJ to segment greyscale image into black and white. The hypoechoic fraction of the nerve will be measured as a fraction of black in percent of the nerve. Measures of echogenicity will be performed bilaterally. The mean value of the right and left nerves will be used for analysis.(13) Echogenicity is classified into three classes: 1. hypoechogenic (fraction of black > 67%), 2. mixed hypo-/hyperechogenic (fraction of black 33–67%) and 3. hyperechogenic (fraction of black < 33%).(14)

*Conditioned Pain Modulation*. Cold pressor test (CPT) is a psychophysical test used to assess the conditioned pain modulation (CPM), where the cold water (between 1ºC and 4ºC) is the pain conditioning stimulus, and pressure pain threshold (PPT) is the test stimulus. The CPT is an appropriate method to assess the descending nociceptive inhibitory system.(15) The participant will be instructed to remain with the hand immersed in water for 1 minute without making muscle contractions or changes in position. The withdrawal from the water will be allowed when the patient could no longer tolerate the painful stimulus. Room temperature, humidity, lighting, and noise will be maintained constant during the entire procedure. The PPT will be performed before and after one minute of the CPT, using a digital pressure algometer (model Force Ten FDX, Wagner Instruments, Greenwich, USA) in the distal part of the dorsal forearm and tibialis anterior muscle. Both sites will be evaluated in the same order for all participants. A familiarisation procedure will be carried out with the pressure algometer by applying pressure to the dominant forearm to ensure that the test had been understood. The force will be gradually increased (1 kilograms-force/s) until the patient pressure sensation changes to pain. The PPT will be registered in kilograms-force (Kgf) when the participant relates the verbal command “pain”. The CPM efficiency classification will be based on the evidence of pain modulation altered in two areas. Only participants with the inefficiency of the CPM in both locations (the anterior tibialis muscle and the distal part of the dorsal forearm) will be classified as impaired pain modulation. Upper and lower limb sites will be used to avoid the inclusion of the participants with peripheral sensitisation according to recommendations for conditioned pain modulation.(16) In addition, the CPM efficiency will be assessed by calculating the difference between the PPT values in CPT (differences between final and initial value). Negative values will represent an inefficiency of the CPM, and null or positive values will be considered a typical CPM response.

*Psychosocial Factors.* Brief Psychological Screening Questions (BPSQ) is a self-reported questionnaire that assesses the influence of psychosocial factors on an individual's health. Anxiety symptoms, social isolation, catastrophisation, depression symptoms, kinesiophobia and perceived stress are the domains assessed. The score for each question varies between 0 and 10, with the value 0 (zero) representing "never" and the value 10 (ten) representing "always".(17) The cut-off point for each item was previously established with a value of 5 for symptoms of anxiety, 4 for social isolation, 4 for catastrophisation, 8 for symptoms of depression, 8.5 for kinesiophobia(18) and 7 for perceived stress.(17) In addition, self-efficacy will be evaluated by the isolated item “I am confident I can cope with my condition”. The patient will score each question on a Likert scale between 1 (strongly disagree) and 10 (strongly agree). The higher the score, the higher the level of self-efficacy.(19)

**REFERENCES**

1. Splenger DM. Clinical evaluation of the low back pain region. Nord M, Andersson GBJ, Pope MH Musculoskelet Disord Work Princ Pract St Louis Mosby. 1997;277–87.

2. Collina DD. Quantificação de limiares térmicos em fibras finas. 2012.

3. Freynhagen R, Baron R, Gockel U, Tölle TR. Pain DETECT: a new screening questionnaire to identify neuropathic components in patients with back pain. Curr Med Res Opin. 2006;22(10):1911–20.

4. Freynhagen R, Tölle TR, Gockel U, Baron R. The painDETECT project - Far more than a screening tool on neuropathic pain. Curr Med Res Opin. 2016;32(6):1033–57.

5. Boyd BS. Measurement properties of a hand-held inclinometer during straight leg raise neurodynamic testing. Physiotherapy. 2012;98(2):174–9.

6. Tousignant-Laflamme Y, Boutin N, Dion AM, Vallée C-A. Reliability and criterion validity of two applications of the iPhone^TM^ to measure cervical range of motion in healthy participants. J Neuroeng Rehabil. 2013;10(1):1–9.

7. Boyd BS, Wanek L, Gray AT, Topp KS. Mechanosensitivity of the lower extremity nervous system during straight-leg raise neurodynamic testing in healthy individuals. J Orthop Sport Phys Ther. 2009;39(11):780–90.

8. Martínez MDA, Cubas CL, Girbés EL. Ulnar nerve neurodynamic test: study of the normal sensory response in asymptomatic individuals. J Orthop Sport Phys Ther. 2014;44(6):450–6.

9. Sierra-Silvestre E, Torres Lacomba M, de la Villa Polo P. Effect of leg dominance, gender and age on sensory responses to structural differentiation of straight leg raise test in asymptomatic subjects: a cross-sectional study. J Man Manip Ther. 2017;25(2):91–7.

10. Montaner-Cuello A, Bueno-Gracia E, Bueno-Aranzabal M, Borrella-Andrés S, López-de-Celis C, Malo-Urriés M. Normal response to sural neurodynamic test in asymptomatic participants. A cross-sectional study. Musculoskelet Sci Pract. 2020;50:102258.

11. Coppieters MW, Andersen LS, Johansen R, Giskegjerde PK, Høivik M, Vestre S, et al. Excursion of the sciatic nerve during nerve mobilization exercises: an in vivo cross-sectional study using dynamic ultrasound imaging. J Orthop Sport Phys Ther. 2015;45(10):731–7.

12. Ellis R, Hing W, Dilley A, McNair P. Reliability of measuring sciatic and tibial nerve movement with diagnostic ultrasound during a neural mobilisation technique. Ultrasound Med Biol. 2008;34(8):1209–16.

13. Fisse AL, Pitarokoili K, Motte J, Gamber D, Kerasnoudis A, Gold R, et al. Nerve echogenicity and intranerve CSA variability in high-resolution nerve ultrasound (HRUS) in chronic inflammatory demyelinating polyneuropathy (CIDP). J Neurol [Internet]. 2019;266(2):468–75. Available from: http://dx.doi.org/10.1007/s00415-018-9158-3

14. Padua L, Granata G, Sabatelli M, Inghilleri M, Lucchetta M, Luigetti M, et al. Heterogeneity of root and nerve ultrasound pattern in CIDP patients. Clin Neurophysiol [Internet]. 2014;125(1):160–5. Available from: http://dx.doi.org/10.1016/j.clinph.2013.07.023

15. Lewis GN, Heales L, Rice DA, Rome K, McNair PJ. Reliability of the conditioned pain modulation paradigm to assess endogenous inhibitory pain pathways. Pain Res Manag. 2012;17(2):98–102.

16. Yarnitsky D, Bouhassira D, Drewes AM, Fillingim RB, Granot M, Hansson P, et al. Recommendations on practice of conditioned pain modulation (CPM) testing. Eur J Pain (United Kingdom). 2015;19(6):805–6.

17. Vaegter HB, Handberg G, Kent P. (345) Brief psychological screening questions can be useful for ruling out psychological conditions in patients with chronic pain. J Pain. 2017;18(4):S61.

18. Kent P, Mirkhil S, Keating J, Buchbinder R, Manniche C, Albert HB. The concurrent validity of brief screening questions for anxiety, depression, social isolation, catastrophization, and fear of movement in people with low back pain. Clin J Pain. 2013/11/28. 2014;30(6):479–89.

19. Wertli MM, Held U, Lis A, Campello M, Weiser S. Both positive and negative beliefs are important in patients with spine pain: findings from the Occupational and Industrial Orthopaedic Center registry. Spine J. 2018;18(8):1463–74.
