# Supplementary material for: Neural management plus advice to stay active on clinical measures and sciatic neurodynamic for patients with chronic sciatica: Study protocol for a controlled randomised clinical trial
Source: PLoS One. 2022 Feb 4;17(2):e0263152. doi: 10.1371/journal.pone.0263152 (PMC8815873; doi:10.1371/journal.pone.0263152)
Supplement: S3 File — (PDF) [file pone.0263152.s003.pdf]

[Logo of Plataforma Brasil]

MINISTRY OF HEALTH - National Health Council - National Research Ethics Commission – CONEP
RESEARCH PROJECT INVOLVING HUMAN BEINGS

Research Project:
Neural management plus advice to stay active on clinical measures and sciatic neurodynamic in patients with chronic sciatica: a controlled randomised clinical trial.

Preliminary Information

------ Main Responsible --

CPF/Document: 144.542.877-67	Name: LETICIA AMARAL CORRÊA
Telephone: 21979100168	Email: leticia.amaral@live.com

------ Proposing Institution --

CNPJ: 34.008.227/0001-03	Institution Name: AUGUSTO MOTTA UNIFIED EDUCATION SOCIETY [SOCIEDADE UNIFICADA DE ENSINO AUGUSTO MOTTA]

Is it an international study? No

- **Assistants**

CPF/Document	Name
073.393.767-55	Leandro Alberto Calazans Nogueira
149.633.837-56	JULIANA VALENTIM BITTENCOURT

- **Research Team**

CPF/Document	Name
016.279.491-60	ALANNA MARTINS SOARES DE PALMA
149.633.837-56	JULIANA VALENTIM BITTENCOURT
144.032.657-66	JESSICA PINTO MARTINS DO RIO
121.215.867-92	GUSTAVO FELICIO TELLES
876.971.577-72	MARIA ALICE MAINENTI PAGNEZ
073.393.767-55	Leandro Alberto Calazans Nogueira

Study Area

Major Areas of Knowledge (CNPq)

- Major Area 4. Health Sciences

Main Purpose of the Study (WHO)

- Clinical

Research Public Title: Neural management plus advice to stay active in patients with chronic sciatica.

------ Public Contact --

CPF/Document	Name	Telephone	Email
144.542.877-67	LETICIA AMARAL CORRÊA	21979100168	leticia.amaral@live.com

Scientific Contact: Leandro Alberto Calazans Nogueira

Study Design / Financial Support
Study Design: Intervention/Experimental

------ Health conditions or problems --

Health condition or Problem
Chronic sciatica

------ General Descriptors for Health Conditions --
ICD1-10: International Classification of Diseases

ICD Code	ICD Description
M54.5	Low back pain

DeCS: Health Science Descriptors

DECS Code	DECS Description
30665	Low back pain

------ Specific Descriptors for Health Conditions --
ICD1-10: International Classification of Diseases

ICD Code	ICD Description
M54.3	Sciatica
M54.4	Lumbago with sciatica

DeCS: Health Science Descriptors

DECS Code	DECS Description
12966	Sciatica

Type of Intervention: Experimental

Nature of the Intervention
- Other Physiotherapy

------ Intervention Descriptors --
Intervention Descriptors

Interventions
Manual Therapy
Health Education

ICD list

ICD Code	ICD Description
Z50	Care involving use of rehabilitation procedures
Z50.1	Other physical therapy

DECS list

DECS Code	DECS Description
36319	Physical Therapy Specialty

Phase
- Does not apply to the study

Design:
A superiority, blinded, parallel, randomised controlled clinical trial with two arms randomised to 1:1 will be conducted in accordance with the Consolidated Standards of Reporting Trials (CONSORT) and Standard Protocol Items: Recommendations for Interventional Trials (SPIRIT).

------ Financial Support ---

CNPJ	Name	Email	Telephone	Type
				Own Financing

------ Keyword --

Keyword
Low Back Pain
Sciatica
Chronic Pain
Manual Therapy
Health Education

Study Detail

Abstract:

Introduction: Low back pain is the main cause of years lived with disability worldwide. Patients with sciatica present a worse prognosis when compared to those with localised low back pain. The main treatment strategy available in the literature for these patients is the advice to stay active. Other conservative treatments, such as neural management, can contribute to a significant sciatica recovery. However, the effects of neural management in patients with sciatica have not yet been robustly evaluated in the literature. Objective: To compare the effects of adding neural management to advice to stay active versus only advice to stay active in improving pain intensity, functional limitation, and sciatic neurodynamic of patients with chronic sciatica. Secondarily, the study aims to assess the relationship between the characteristics of the participants with the self-reported pain intensity. Ultimately, we will analyse whether the effect of the treatment on pain intensity and functional limitation is mediated by sociodemographic factors, pain characteristics, and psychosocial aspects. Methods: A superiority clinical trial, randomised with 1:1 allocation, controlled, with a parallel-group and blind examiner will be conducted with 210 participants with chronic sciatica and aged between 18 and 65 years old, with a pain intensity higher than 3 on the Numerical Pain Rating Scale. Patients will be recruited at the outpatient clinics of Augusto Motta University Centre (UNISUAM) and from community announcements. The experimental group will receive soft tissue mobilisation techniques and neural mobilisation techniques for 30 minutes per weekly session, in 10 sessions, as well as advice to stay active in your daily activities, information about physical activity, imaging exams and sciatica, during 5 sessions lasting 25-30 minutes, biweekly. The control group will receive only the advice to stay active protocol. Revaluations will be performed on 5, 10, and 26 weeks after randomisation. The primary outcomes will be pain intensity and functional limitation. Secondary outcomes will include the presence of neuropathic symptoms, sciatic neurodynamic, conditioned pain modulation, and psychosocial factors. Adverse events and patient satisfaction will also be assessed. Comparisons between groups will be performed by the mixed linear regression model following the intention-to-treat analysis. Secondarily, a multivariate linear regression model will be implemented to assess the relationship between the characteristics of patients with chronic sciatica and self-reported pain intensity. Finally, the evaluation of the mediators will be performed through simple mediation analysis, considering one variable per model. Expected results: It is expected that patients included in the experimental group present a significant reduction in pain intensity, improved functional limitation and sciatic neurodynamic after treatment when compared to the control group. Analysis of the participant's baseline characteristics will provide information about the most relevant aspects of the participant's self-reported pain perception. Furthermore, the analysis of mediators will identify potential factors that explain the clinical improvement of the participant with chronic sciatica submitted to the study intervention.

Introduction:

Low back pain is the leading cause of disability worldwide (James et al., 2018; Marinho et al., 2018; Wu et al., 2020). In primary care, approximately 60% of patients with low back pain report leg pain (Hill et al., 2011). Sciatica is a lumbosacral radiculopathy presented as a severe form of low back pain characterised by radiating leg pain and is considered chronic when symptoms last more than 12 weeks (Ropper and Zafonte, 2015). Patients with sciatica have a worse prognosis, higher incidence of disability compensation, in addition to higher levels of pain and functional limitation when compared to patients with localised low back pain (Hartvigsen et al., 2018; Jensen et al., 2019; Konstantinou et al., 2013; Kristman et al., 2012). The success of the treatment is dependent on various identified prognostic factors, such as sociodemographic factors (e.g. male), clinical (e.g. longer duration of low back pain, comorbidities), lifestyle (e.g. smoker), and psychosocial factors (e.g. kinesiophobia) (Haugen et al., 2012). These prognostic factors are important considerations to identify appropriate treatment and better management of patients with sciatica. Clinical practice guidelines and systematic reviews recommend several conservative treatments as the initial treatment for patients with sciatica (Bernstein et al., 2017; Campos, de, 2017). Conservative treatment strategies aim to reduce pain intensity and maintain the function of patients. Evidence presents that active conservative treatment is effective in patients with severe sciatica (Albert and Manniche, 2012) and advice to stay active continues to be a first-line recommendation for the management of sciatica in most international clinical practice guidelines (National Guideline Center UK, 2016; Stochkendahl et al., 2018). Other conservative strategies, such as exercise, has been found not to be superior to advice to stay active in reducing pain intensity and disability of patients with sciatica (Fernandez et al., 2015). In physiotherapy, advice to stay active or physical exercise presents little or no difference for patients with sciatica (Dahm et al., 2010). However, neurodynamic treatment has shown a significant reduction of leg pain intensity and improved function after four weeks of intervention in patients with chronic nerve-related leg pain (Ferreira et al., 2016). Despite this, conservative treatments such as advice to stay active and neural management are strategies that should be implemented to treat patients with sciatica. There is growing evidence that neural management may facilitate recovery in patients with sciatica. Neural management aims to restore peripheral nervous system homeostasis through nerve and surrounding tissue mobilisation (Coppieters et al., 2015; Coppieters, Hough and Dilley, 2009). Neural management may assist in the restoration of the neurodynamic of the sciatic nerve. Compression of the sciatic nerve may be associated with changes in nerve function (Ropper and Zafonte, 2015). Besides, the improvement of clinical symptoms in sciatica patients is related to the normalisation of nerve mobility (Pesonen et al., 2019). A decrease in intraneural oedema and improvement in temporal summation and median nerve latency were presented after neural management for different conditions (e.g. nerve-related neck and arm pain, tarsal tunnel syndrome, and plantar heel pain) (Basson et al., 2017). Neural involvement may be investigated by ultrasound imaging. The use of ultrasound images to assess the properties of nerves is becoming popular (Ellis et al., 2018). Ultrasound images quantify the nerve neurodynamic, including excursion and the cross-sectional area of the nerve (Beekman and Visser, 2004; Dilley et al., 2001; Silva et al., 2014). Thus, ultrasound images represent an exam able to assess the effect of neural management treatment on sciatic nerve structure. Currently, the benefits of neural management as a treatment strategy in patients with chronic sciatica are limited, as the available evidence is of low quality. Neural management has shown positive results for improving pain intensity and function in patients with low back pain (Almeida et al., 2019; Basson et al., 2017; Neto et al., 2017) and has been presented as superior to other treatments such as physical exercise, conventional physiotherapy, traction

or manual therapy in patients with chronic nerve-related musculoskeletal pain (e.g. sciatica, chronic radicular low back pain, nerve-related neck and arm pain, and carpal tunnel syndrome) (Su and Lim, 2016). Previous studies using neurodynamic treatment presented methodological limitations as high population heterogeneity (Neto et al., 2017), small sample size (Almeida et al., 2019; Ferreira et al., 2016), and low-quality of evidence (Basson et al., 2017; Neto et al., 2017). Despite being a frequently used strategy, there is a lack of high-quality evidence to demonstrate the clinical effects and benefits of neural management for patients with chronic sciatica. Therefore, we propose a randomised controlled clinical trial to compare the effects of adding neural management to advice to stay active versus advice to stay active alone in improving clinical measures and in the sciatic neurodynamic in patients with chronic sciatica. Secondarily, the study aims to assess the relationship between the participants' characteristics with self-reported pain intensity. Ultimately, we will analyse whether the treatment effect on pain intensity and functional limitation is mediated by sociodemographic factors, pain characteristics, and psychosocial aspects.

Hypothesis:

The hypothesis of the present study is that patients with chronic sciatica allocated in the neural management added to advice to stay active group present a statistically significant reduction in pain intensity, improvement in functionality, and improvement of the sciatic neurodynamic (e.g. a greater decrease in the cross-sectional area of the sciatic nerve) after the treatment period (short, medium, and long term) superior to patients with chronic sciatica allocated in the group of advice to stay active alone. In addition, we expect that the analysis of the participant's baseline characteristics will provide information about the most relevant aspects in the participant's self-reported pain perception. Finally, the analysis of mediators will make it possible to identify potential factors that explain the clinical improvement of the participant with chronic sciatica submitted to the study interventions.

Primary Objective:

To analyse the effects of neural management added to advice to stay active compared to advice to stay active alone in the medium term (ten weeks after randomisation), improving pain intensity and functionality in patients with chronic sciatica.

Secondary Objective:

1. To analyse the effects of neural management added to advice to stay active compared to advice to stay active alone in the medium term (ten weeks after randomisation) in sciatic neurodynamic of patients with chronic sciatica. 2. To assess the relationship between sociodemographic characteristics (gender, education level, and family income), pain characteristics (pain areas, pain duration, presence of neuropathic symptoms, and conditioned pain modulation), sciatic neurodynamic (cross-sectional area of the sciatic nerve, nerve-to-skin distance, echogenicity index, and hip range of motion during the straight leg raise test), and psychosocial factors (symptoms of anxiety, social isolation, catastrophising, symptoms of depression, kinesiophobia, perceived stress, and self-efficacy) and the intensity of self-reported pain in participants with chronic sciatica. 3. To analyse the effects of neural management added to advice to stay active compared to advice to stay active alone in the short term (five weeks after randomisation) and long term (twenty-six weeks after randomisation) to improve pain intensity, functionality, and sciatic neurodynamic in patients with chronic sciatica. 4. Identify potential outcome mediators among variables of sociodemographic factors (gender, education level, and family income), pain characteristics (pain areas, pain duration, presence of neuropathic symptoms, and conditioned pain modulation), and psychosocial factors (symptoms of anxiety, social isolation, catastrophising, symptoms of depression, kinesiophobia, perceived stress, and self-efficacy).

Proposed Methodology:

The experimental group will receive neural management added to advice to stay active, and the control group will receive only the advice to stay active protocol. The experimental protocol will be performed by two trained physiotherapists, blind to the initial assessment (examiner 3 – J.V.B. and examiner 4 – G.T.). It will be conducted in accordance with the Template for Intervention Description and Replication (TIDieR) requirements (Hoffmann et al., 2014).

Advice to stay active

The experimental group and the control group will receive the same advice to stay active protocol. In the first session, all patients will receive a booklet of activities (Appendix 6) developed by the researchers, in which patients will have to register their activities of daily living developed during the week (for instance: sweeping the house, going to the gym, meeting friends, working, among others). The intervention will be conducted at the UNISUAM outpatient clinic, and there will be no modification of the protocol during the study.

The intervention will be performed by a physiotherapist (examiner 3 – J.V.B.) with 2 years of clinical experience. All participants will receive as intervention the advice to stay active in their daily living activities, information on physical activities, imaging tests, and information about sciatica, fortnightly for 5 sessions performed in person and individually, lasting approximately 25-30 minutes each session (Pico-Espinosa et al., 2020). Advice to stay active will focus on two themes: (1) Harmful effects of pain and (2) Beneficial effects on pain (Ferreira et al., 2016). In the harmful effects of pain, we will discuss avoidance of activities of daily living (Ferreira et al., 2016; Malmivaara et al., 1995; Vroomen et al., 1999), prolonged rest (Ferreira et al., 2016), sedentary lifestyle, and imaging exams (Bernstein et al., 2017; Oliveira et al., 2018). In the beneficial effects for pain, we will discuss the benefits of staying active (Dahm et al., 2010; Damsgård et al., 2011; Fernandez et al., 2015; Huber et al., 2011; Oliveira et al., 2018; Paatelma et al., 2008), how to avoid bed rest and the positive aspects of returning to work (Oliveira et al., 2018; Paatelma et al., 2008; Waddell, Feder, and Lewis, 1997), physical activity practice (Chou et al., 2017; Oliveira et al., 2018; Paatelma et al., 2008) and understanding of sciatica, pain tolerance, and favourable prognosis (Fernandez et al., 2015; Paatelma et al., 2008). In the first four sessions of advice to stay active, one harmful effect topic and one beneficial effect topic will be addressed, and at the last session, participants will receive a summary of the content covered in all previous sessions.

Neural management

Neural management will be performed by a physical therapist (G.T.) with 9 years of clinical experience. Participants will receive neural mobilisation techniques and soft tissue mobilisation techniques for 30 minutes each weekly session, in 10 sessions, considering the participant availability. All patients randomised to this treatment group will be treated by the same physical therapist, submitted to the same protocol, and there will be no modification of the protocol during the study. Treatment sessions will be conducted at the UNISUAM outpatient clinic.

In each neural management session, participants will receive the following protocol in the affected lower limb, based on a previous study by our research group (Almeida et al., 2019).

Inclusion Criteria:

Potential participants will be assessed by a physiotherapist (examiner 1 – M.A.P.) to confirm the diagnosis of sciatica (Appendix 3). Thus, participants aged between 18 and 65 years who present chronic sciatica with moderate or severe leg pain intensity (Numerical Pain Rating Scale score greater than 3) will be included (Palos et al., 2006; Stynes et al., 2018). The inclusion criteria will also include the presence of higher pain intensity in the lower limb compared to the area

of the lower back, being symptomatic at the time of evaluation and clinical evaluation to confirm the diagnosis of chronic sciatica.
The clinical diagnosis of sciatica will be confirmed in case the participant presents two or more positive findings in the neurological evaluation. The clinical criteria will be based on a recent study that presented a reference standard for the sciatica diagnosis based on 5 items: positive neural tension in the sciatic nerve, neurological deficit, pain below the knee, higher pain intensity in the leg compared to the lower back, and subjective sensory changes (Stynes et al., 2018). Chronic sciatica will then be defined as pain radiating unilaterally to the lower limb below the knee, with sensory symptoms involving the dermatomes of L4 to S1, in addition to possible neurological symptoms for more than 3 months (Jensen et al., 2019; Ropper and Zafonte, 2015; Valat et al., 2010).

Exclusion criteria:

Participants will be excluded if they are undergoing any treatment for sciatica, or if they plan to undergo any other procedure for sciatica in the study period; present signs of serious spinal pathology (cauda equine syndrome, trauma, fractures, or cancer); history of surgery in the lumbar spine and lower limb in the last year; history of symptomatic leg or abdominal surgery in the past year; rheumatologic disease in the acute inflammatory phase; if they are receiving disability compensation due to low back pain; pregnant or puerperal women who are breastfeeding during the assessment and/or treatment period; self-reported psychiatric diagnosis; history of cancer or cancer-related pain.

Risks:

Participants will answer the questionnaires and perform the proposed evaluations. The application of questionnaires presents minimal risks related to the embarrassment of not knowing how to answer any item. Participants will be able to complete the questionnaire in the time they deem necessary and may leave items unanswered if they are unable to respond. The responses of participants will be kept confidential. The assessment of Conditioned Pain Modulation (CPT) may cause mild or moderate transiently pain and will be performed in a maximum of 1 minute. If there is any discomfort during the procedures, the research examiners will wait the necessary period with the patient for the patient to return to his/her pre-test sensation. The study researchers will provide the appropriate treatment or referral if the discomfort does not disappear. The use of neural management as a treatment for clinical variables has an immediate effect and no evidence of detrimental effects to the participant (Nee et al., 2012). Thus, the present study presents minimal risk to study participants and the research team.

Benefits:

This project has considerable relevance due to the high social impact generated by the offer of free service and subsequent follow-up to the population of the metropolitan area of Rio de Janeiro who suffers from chronic sciatica. In addition, both groups are expected to present clinical improvements in your condition, as treatment considered the gold standard will be offered (advice to stay active) for patients with sciatica. It is also expected to contribute to the evidence that makes available the use of neural management plus advice to stay active in clinical practice and future research.

Data Analysis Methodology:

All data will be stored in a Microsoft Excel spreadsheet (Microsoft Corporation) and made available for statistical analyses. Data analysis will be performed by intention-to-treat using multiple imputation technique for missing data analysis and conducted by an independent and blinded examiner. The reasons for missing data will be reported. The demographic and clinical variables of the study population will be presented as mean and standard deviation for continuous variables. Categorical variables will be presented as percentages. For continuous variables, the normal distribution of the study outcomes will be verified by the Shapiro-Wilk test. A significance level of less than 5% ($P < 0.05$) will be considered for all analyses. The statistical analysis will be performed using JASP version 0.10.2.0, and graph analysis will be performed using GraphPad Prism software (GraphPad Software version 7.00 for MacBook, San Diego, CA, USA). Primary analysis: The between-group differences for the primary outcomes considering the change in pain intensity and functionality from baseline to each 5, 10- and 26-weeks after randomisation will be calculated using Mixed Linear Models using the interaction terms of treatment group versus time. The pain intensity and functionality will be investigated in separate models. Secondary analysis: The between-group differences for the secondary outcome considering the change in neuropathic symptoms, sciatic neurodynamic, conditioned pain modulation, and psychosocial factors from baseline to each 5, 10- and 26-weeks after randomisation will be calculated using Mixed Linear Models using the interaction terms of treatment group versus time. A multivariate linear regression model will be implemented to assess the relationship between clinical characteristics of patients with chronic sciatica and self-reported pain intensity. Sociodemographic characteristics (e.g. age, weight, height, body mass index, sex, among others) and clinical characteristics (e.g. pain intensity, pain areas, pain duration, disability level, presence of neuropathic symptoms, among others) will be tested in the multivariate analysis. The variables that satisfy the cut-off point $p<0.1$ in univariate analyses will be incorporated as covariates in the multivariate analysis. The clinical characteristics and the sciatic neurodynamic will be considered as dependent variables. Tertiary analysis: The patients' adherence, serious adverse events or adverse events, and patient satisfaction will be described and presented as mean and standard deviation for continuous variables and as absolute values and percentages for categorical variables. The mediation analysis will be performed to assess whether sociodemographic factors (sex, educational level, and family income), pain characteristics (pain intensity, pain areas, and pain duration), and psychosocial factors (symptoms of anxiety, social isolation, catastrophising, symptoms of depression, kinesiophobia, perceived stress, and self-efficacy) affect the primary outcomes. The mediator's analysis will be performed by simple mediation analysis, considering one variable per model, and we will use data collected at baseline. Models will be designed separately for pain intensity and functional limitation, considering the treatment group as independent variable (X), pain intensity and functional limitation as dependent variable (Y), and sociodemographic factor, pain characteristics, and psychosocial factors as mediators (M).

Primary Outcome:

Pain intensity - Pain intensity will be measured using the Numeric Pain Rating Scale (NPRS). The NPRS varies between 0 (no pain) and 10 (worst pain possible). Participants will be asked to rate their leg pain and low back pain at the evaluation moment. The NPRS is a common scale in pain studies, including sciatica and has good reproducibility levels (Hawker et al., 2011). The minimally clinically important difference considered in the current study will be an effect size of 0.2. The duration of pain will be recorded in months. Functional limitation - Functional limitation will be investigated using the Patient-Specific Functional Scale (PSFS). The PSFS is a self-reported measure widely used in various musculoskeletal conditions, with excellent reproducibility (Maughan and Lewis, 2010). Participants will be asked to identify three important activities they present functional limitations due to their health condition, in the case of this study, due to chronic sciatica. Each activity is classified between 0 (unable to perform the activity) and 10 (able to perform the activity at the level before injury). The final score is calculated from the mean of the activities reported, ranging between 0 and 10, in which a lower score identifies greater functional disability (Kowalchuk Horn et al., 2012).

Secondary Outcome:

Presence of Neuropathic Symptoms - The presence of neuropathic symptoms will be assessed by a clinical examination for neurological assessment and the painDETECT questionnaire (Appendix 1). Clinical examination for neurological assessment will include testing for muscle weakness in L5/S1 myotomes, leg perimetry, and sensitivity in corresponding dermatomes. The assessment of muscle weakness will include two tests (walking on the heels and walking on the toes) (Splenger, 1997). Perimetry will be performed bilaterally in the middle region of the leg (15 cm below the lower edge of the patella) using a tape measure. The sensitive neurological examination will be performed bilaterally, the patient will be in the dorsal decubitus position and eyes closed, and composed by examining vibratory, painful, pressure, and thermal sensitivity. The painDETECT also will be used to assess the presence of neuropathic symptoms (Freynhagen et al., 2006, 2016).
Sciatic neurodynamic – Sciatic neurodynamic will be assessed as previously performed by our research group (Pagnez et al., 2019) through the cross-sectional area of the sciatic nerve, nerve-skin distance, echogenicity index, and hip range of motion during the straight leg raise test. The cross-sectional area of the sciatic nerve, nerve-skin distance, and echogenicity index will be measured using ultrasound (Mobissom, portable MDuo model, São Paulo – Brazil) with a double transducer. The hip range of motion during the straight leg raise test.

Conditioned Pain Modulation - The Cold Pressor Test (CPT) is a psychophysical test used to assess the conditioned pain modulation (CPM), where the cold water (between 1ºC and 4ºC) is the pain conditioning stimulus of pain and the pressure pain threshold (PPT) is the test stimulus.

Psychosocial Factors – – The Brief Psychological Screening Questions (BPSQ) is a self-reported questionnaire that assesses the influence of psychosocial factors on an individual's health.

Sample Size in Brazil: 210

------ Recruitment Countries --

Study Origin Country	Country	Number of research participants
Yes	BRAZIL	210

Other information

Will there be the use of secondary data sources (medical records, demographics, etc.)?
No

Inform the number of individuals personally approached, recruited, or who will undergo any intervention in this research centre:
210

Groups in which research participants will be divided at this centre

Group ID	Number of Individuals	Intervention to be performed
Control group	105	Advice to stay active
Experimental group	105	Neural management added to advice to stay active

Is the Study Multicentric in Brazil?
No

Does it propose exemption from the ICF [Informed Consent Form]?
No

Will there be retention of samples for storage in a bank?
No

Execution schedule

Stage identification	Start (DD/MM/AAAA)	End (DD/MM/AAAA)
REBEC registration	20/01/2021	31/01/2021
Manuscript preparation	20/01/2023	10/06/2023
Data analysis	10/11/2022	10/12/2022
Research protocol submission	15/03/2021	15/06/2021
Thesis write up	30/06/2023	30/09/2023
Adjustments requested by the examining committee	21/11/2023	20/12/2023
Delivery of the final version of the thesis	20/12/2023	30/12/2023
Data collection and tabulation	30/03/2021	10/10/2022
REC submission	30/10/2020	18/12/2020

Manuscript submission	20/12/2023	31/12/2023
Thesis defence	01/11/2023	20/11/2023

Financial Budget

Budget Identification	Type	Value in Reais (R$)
Brown envelopes	Cost	R$ 61.50
Black ink cartridge	Cost	R$ 80.00
Ballpoint pen	Cost	R$ 140.00
Red pilot pen	Cost	R$ 200.00
Printing ink	Cost	R$ 50.00
Information folder	Cost	R$ 450.00
A4 sheets of paper	Cost	R$ 198.00
Total in R$		R$ 1179.50

Bibliography:

ALBERT, H. B.; MANNICHE, C. The efficacy of systematic active conservative treatment for patients with severe sciatica: A single-blind, randomized, clinical, controlled trial. Spine, v. 37, n. 7, p. 531–542, 2012. ALMEIDA, R. S. DE et al. Pragmatic neural tissue management improves short-term pain and disability in patients with sciatica: a single-arm clinical trial. Journal of Manual & Manipulative Therapy, v. 27, n. 4, p. 208–214, 2019. BAGG, M. K. et al. The RESOLVE Trial for people with chronic low back pain: statistical analysis plan. Brazilian Journal of Physical Therapy, v. S1413-3555, n. 20, p. 30188– X, 2020. BASSON, A. et al. The effectiveness of neural mobilization for neuromusculoskeletal conditions: a systematic review and meta-analysis. journal of orthopaedic & sports physical therapy, v. 47, n. 9, p. 593–615, 2017. BEEKMAN, R.; VISSER, L. H. High resolution sonography of the peripheral nervous system–a review of the literature. European Journal of Neurology, v. 11, n. 5, p. 305–314, 2004. BERNSTEIN, I. A. et al. Low back pain and sciatica: summary of NICE guidance. BMJ, v. 356, p. i6748, 2017. CAMPOS, T. F. DE. Low back pain and sciatica in over 16s: assessment and management NICE Guideline [NG59]. Journal of Physiotherapy, v. 63, n. 2, p. 120, 2017. CHAN, A. W. et al. Spirit 2013 statement: Defining standard protocol items for clinical trials. Chinese Journal of Evidence-Based Medicine, v. 13, n. 12, p. 1501–1507, 2013. CHOU, R. et al. Nonpharmacologic therapies for low back pain: a systematic review for an American College of Physicians clinical practice guideline. Annals of internal medicine, v. 166, n. 7, p. 493–505, 2017. COLLINA, D. D. Quantificação de limiares térmicos em fibras finas. [s.l: s.n.]. COPPIETERS, M. W. et al. Excursion of the sciatic nerve during nerve mobilization exercises: an in vivo cross-sectional study using dynamic ultrasound imaging. Journal of orthopaedic & sports physical therapy, v. 45, n. 10, p. 731–737, 2015. COPPIETERS, M. W.; HOUGH, A. D.; DILLEY, A. Different nerve-gliding exercises induce different magnitudes of median nerve longitudinal excursion: an in vivo study using dynamic ultrasound imaging. journal of orthopaedic & sports physical therapy, v. 39, n. 3, p. 164–171, 2009. DAHM, K. T. et al. Advice to rest in bed versus advice to stay active for acute low back pain and sciatica. Cochrane database of systematic reviews, n. 6, p. CD007612, 2010. DAMSGÅRD, E. et al. Staying active despite pain: Pain beliefs and experiences with activity related pain in patients with chronic musculoskeletal pain. Scandinavian Journal of Caring Sciences, v. 25, n. 1, p. 108–116, 2011. DHONDT, E. et al. Predicting treatment adherence and outcome to outpatient multimodal rehabilitation in chronic low back pain. Journal of Back and Musculoskeletal Rehabilitation, v. 33, n. 2, p. 277–293, 2020. DILLEY, A. et al. The use of cross-correlation analysis between high-frequency ultrasound images to measure longitudinal median nerve movement. Ultrasound in medicine & biology, v. 27, n. 9, p. 1211–1218, 2001. ELLIS, R. et al. Reliability of measuring sciatic and tibial nerve movement with diagnostic ultrasound during a neural mobilisation technique. Ultrasound in medicine & biology, v. 34, n. 8, p. 1209–1216, 2008. ___. Ultrasound Elastographic measurement of sciatic nerve displacement and shear strain during active and passive knee extension. Journal of Ultrasound in Medicine, v. 37, n. 8, p. 2091–2103, 2018. FERNANDEZ, M. et al. Advice to Stay Active or Structured Exercise in the Management of Sciatica. Spine, v. 40, n. 18, p. 1457–1466, 2015. FERREIRA, G. et al. Neurodynamic treatment did not improve pain and disability at two weeks in patients with chronic nerve-related leg pain: a randomised trial. Journal of physiotherapy, v. 62, n. 4, p. 197–202, 2016. FISSE, A. L. et al. Nerve echogenicity and intra nerve CSA variability in high resolution nerve ultrasound (HRUS) in chronic inflammatory demyelinating polyneuropathy (CIDP). Journal of Neurology, v. 266, n. 2, p. 468–475, 2019. FREYNHAGEN, R. et al. Pain DETECT: a new screening questionnaire to identify neuropathic components in patients with back pain. Current medical research and opinion, v. 22, n. 10, p. 1911–1920, 2006. ___. The painDETECT project - Far more than a screening tool on neuropathic pain. Current Medical Research and Opinion, v. 32, n. 6, p. 1033–1057, 2016. FROUD, R. et al. The Power of Low Back Pain Trials: A Systematic Review of Power, Sample Size, and Reporting of Sample Size Calculations Over Time, in Trials Published Between 1980 and 2012. Spine (Phila Pa 1976), v. 42, n. 11, p. E680–E686, 2017. HARTVIGSEN, J. et al. What low back pain is and why we need to pay attention. The Lancet, v. 391, n. 10137, p. 2356–2367, 2018. HAUGEN, A. J. et al. Prognostic factors for non-success in patients with sciatica and disc herniation. BMC musculoskeletal disorders, v. 13, n. 1, p. 183, 2012. HAWKER, G. A. et al. Measures of adult pain: Visual Analog Scale for Pain (VAS Pain), Numeric Rating Scale for Pain (NRS Pain), McGill Pain Questionnaire (MPQ), Short-Form McGill Pain Questionnaire (SF-MPQ), Chronic Pain Grade Scale (CPGS), Short Form-36 Bodily Pain Scale (SF. Arthritis care & research, v. 63, n. S11, p. S240–S252, 2011. HILL, J. C. et al. Clinical outcomes among low back pain consulters with referred leg pain in primary care. Spine, v. 36, n. 25, p. 2168–2175, 2011. HOFFMANN, T. C. et al. Better reporting of interventions: Template for intervention description and replication (TIDieR) checklist and guide. BMJ (Online), v. 348, p. g1687, 2014. HUBER, J. et al. The effect of early isometric exercises on clinical and neurophysiological parameters in patients with sciatica: An interventional randomized single blinded study. Isokinetics and Exercise Science, v. 19, n. 3, p. 207–214, 2011. JAMES, S. L. et al. Global, regional, and national incidence, prevalence, and years lived with disability for 354 Diseases and Injuries for 195 countries and territories, 1990-2017: A systematic analysis for the Global Burden of Disease Study 2017. The Lancet, v. 392, n. 10159, p. 1789–1858, 2018. JENSEN, R. K. et al. Diagnosis and treatment of sciatica. BMJ, v. 367, p. l6273, 2019. KENT, P. et al. The concurrent validity of brief screening questions for anxiety, depression, social isolation, catastrophization, and fear of movement in people with low back pain. The Clinical Journal of Pain, v. 30, n. 6, p. 479–489, 2014. KONSTANTINOU, K. et al. The impact of low back-related leg pain on outcomes as compared with low back pain alone: a systematic review of the literature. The Clinical journal of pain, v. 29, n. 7, p. 644–654, 2013. KOWALCHUK HORN, K. et al. The patient-specific functional scale: Psychometrics, clinimetrics, and application as a clinical outcome measure. Journal of Orthopaedic and Sports Physical Therapy, v. 42, n. 1, p. 30–42, 2012. KRISTMAN, V. L. et al. Does radiating spinal pain determine future work disability? A retrospective cohort study of 22,952 Danish twins. Spine, v. 37, n. 11, p. 1003–1013, 2012. LEWIS, G. N. et al. Reliability of the conditioned pain modulation paradigm to assess endogenous inhibitory pain pathways. Pain Research and Management, v. 17, n. 2, p. 98–102, 2012. MALMIVAARA, A. et al. The treatment of acute low back pain—bed rest, exercises, or ordinary activity? New England Journal of Medicine, v. 332, n. 6, p. 351–355, 1995. MARINHO, F. et al. Burden of disease in Brazil, 1990–2016: a systematic subnational analysis for the

Global Burden of Disease Study 2016. The Lancet, v. 392, n. 10149, p. 760–775, 1 set. 2018. MAUGHAN, E. F.; LEWIS, J. S. Outcome measures in chronic low back pain. European Spine Journal, v. 19, n. 9, p. 1484–94, 2010. NATIONAL GUIDELINE CENTRE UK. Low Back Pain and Sciatica in Over 16s: Assessment and Management. [s.l.] London: National Institute for Health and Care Excellence (UK), 2016. NEE, R. J. et al. Neural tissue management provides immediate clinically relevant benefits without harmful effects for patients with nerve-related neck and arm pain: a randomised trial. Journal of physiotherapy, v. 58, n. 1, p. 23–31, 2012. NETO, T. et al. Effects of lower body quadrant neural mobilization in healthy and low back pain populations: a systematic review and meta-analysis. Musculoskeletal Science and Practice, v. 27, p. 14–22, 2017. OLIVEIRA, C. B. et al. Clinical practice guidelines for the management of non-specific low back pain in primary care: an updated overview. European Spine Journal, v. 27, n. 11, p. 2791–2803, 2018. OLIVEIRA, N. DE F. C. et al. Measurement properties of the Brazilian Portuguese version of the MedRisk instrument for measuring patient satisfaction with physical therapy care. Journal of orthopaedic & sports physical therapy, v. 44, n. 11, p. 879–889, 2014. PAATELMA, M. et al. Orthopaedic manual therapy, McKenzie method or advice only for low back pain in working adults: a randomized controlled trial with one year follow-up. Journal of rehabilitation medicine, v. 40, n. 10, p. 858–863, 2008. PADUA, L. et al. Heterogeneity of root and nerve ultrasound pattern in CIDP patients. Clinical Neurophysiology, v. 125, n. 1, p. 160–165, 2014. PAGNEZ, M. A. M. et al. The variation of cross-sectional area of the sciatic nerve in flexion-distraction technique: A cross-sectional study. Journal of Manipulative and Physiological Therapeutics, v. 42, n. 2, p. 108–116, 1 fev. 2019. PALOS, G. R. et al. Asking the community about cut points used to describe mild, moderate, and severe pain. J Pain, v. 7, n. 1, p. 49–56, 2006. PESONEN, J. et al. Normalization of Spinal Cord Displacement With the Straight Leg Raise and Resolution of Sciatica in Patients With Lumbar Intervertebral Disc Herniation: A 1.5-year Follow-up Study. Spine, v. 44, n. 15, p. 1064–1077, 2019. PICOESPINOSA, O. J. et al. Deep tissue massage, strengthening and stretching exercises, and a combination of both compared with advice to stay active for subacute or persistent non-specific neck pain: A cost-effectiveness analysis of the Stockholm Neck trial (STONE). Musculoskeletal Science and Practice, v. 46, p. 102109, 2020. ROLKE, R. et al. Quantitative sensory testing in the German Research Network on Neuropathic Pain (DFNS): Standardized protocol and reference values. Pain, v. 123, n. 3, p. 231–243, 2006. ROPPER, A. H.; ZAFONTE, R. D. Sciatica. New England Journal of Medicine, v. 372, n. 13, p. 1240–1248, 2015. SCHULZ, K. F.; ALTMAN, D. G.; MOHER, D. CONSORT 2010 statement: updated guidelines for reporting parallel group randomized trials. Annals of internal medicine, v. 152, n. 11, p. 726–732, 2010. SILVA, A. et al. Quantitative in vivo longitudinal nerve excursion and strain in response to joint movement: a systematic literature review. Clinical Biomechanics, v. 29, n. 8, p. 839–847, 2014. SPLENGER, D. M. Clinical evaluation of the low back pain region. Nordin M, Andersson GBJ, Pope MH. Musculoskeletal disorders in the workplace: principles and practice. St. Louis: Mosby, p. 277–287, 1997. STOCHKENDAHL, M. J. et al. National Clinical Guidelines for non-surgical treatment of patients with recent onset low back pain or lumbar radiculopathy. European Spine Journal, v. 27, n. 1, p. 60–75, 2018. STYNES, S. et al. Clinical diagnostic model for sciatica developed in primary care patients with low back-related leg pain. PloS one, v. 13, n. 4, 2018. SU, Y.; LIM, E. C. W. Does evidence support the use of neural tissue management to reduce pain and disability in nerve-related chronic musculoskeletal pain? The Clinical journal of pain, v. 32, n. 11, p. 991–1004, 2016. VAEGTER, H. B.; HANDBERG, G.; KENT, P. (345) Brief psychological screening questions can be useful for ruling out psychological conditions in patients with chronic pain. The Journal of Pain, v. 18, n. 4, p. S61, 2017. VALAT, J.-P. et al. Sciatica. Best Practice & Research Clinical Rheumatology, v. 24, n. 2, p. 241–252, 2010. VROOMEN, P. C. A. J. et al. Lack of effectiveness of bed rest for sciatica. New England Journal of Medicine, v. 340, n. 6, p. 418–423, 1999. WADDELL, G.; FEDER, G.; LEWIS, M. Systematic reviews of bed rest and advice to stay active for acute low back pain. Br J Gen Pract, v. 47, n. 423, p. 647–652, 1997. WERTLI, M. M. et al. Both positive and negative beliefs are important in patients with spine pain: findings from the Occupational and Industrial Orthopaedic Center registry. Spine J, v. 18, n. 8, p. 1463–1474, 2018. WU, A. et al. Global low back pain prevalence and years lived with disability from 1990 to 2017: estimates from the Global Burden of Disease Study 2017. Annals of Translational Medicine, v. 8, n. 6, 2020. YARNITSKY, D. et al. Recommendations on practice of conditioned pain modulation (CPM) testing. European Journal of Pain (United Kingdom), v. 19, n. 6, p. 805–806, 2015. ZHU, G. C. et al. Concurrent validity of a low-cost and time-efficient clinical sensory test battery to evaluate somatosensory dysfunction. European Journal of Pain (United Kingdom), v. 23, n. 10, p. 1826–1838, 2019.

------ Documents Upload ---

File Attachments:

Type	File
Detailed Project / Investigator Brochure	Projeto_Doutorado_CEP.docx
Schedule	Cronograma.docx
Others	Instrumentos.docx
ICF / Assent Terms / Absence Justification	TCLE.docx
Cover Sheet	FolhaDeRosto_LAC.pdf
Budget	Orcamento.docx
ICF / Assent Terms / Absence Justification	TCLE.docx

Conclude

Keep the entire research project confidential: No

[Logo of UNISUAM].
CENTRO UNIVERSITÁRIO AUGUSTO MOTTA [Augusto Motta University Centre].
[Logo of Plataforma Brasil].

SUBSTANTIATED OPINION OF THE REC

RESEARCH PROJECT DATA

Study title: Neural management plus advice to stay active in patients with chronic sciatica.
Researcher: LETICIA AMARAL CORREA
Subject area:
Version: 1
CAAE [Certificate of Presentation for Ethical Appreciation - Study number]: 40500720.8.0000.5235
Proposing Institution: AUGUSTO MOTTA UNIFIED EDUCATION SOCIETY
Main sponsor: Own financing

OPINION DATA

Opinion Number: 4.457.927

Project presentation:
The doctoral project entitled "Neural management plus advice to stay active on clinical measures and sciatic neurodynamic in patients with chronic sciatica: a controlled randomised clinical trial" is theoretically based that low back pain is the main cause of years lived with disability worldwide. Patients with sciatica present a worse prognosis when compared to those with localised low back pain. The main treatment strategy available in literature for these patients is the advice to stay active. Other conservative treatments, such as neural management, can contribute to a significant sciatica recovery. However, the effects of neural management in patients with sciatica have not yet been robustly evaluated in the literature. Thus, the main objective of this project is the comparison of the effects of adding neural management to advice to stay active versus only advice to stay active in improving pain intensity, functional limitation, and sciatic neurodynamic of patients with chronic sciatica.

Study objective: To compare the effects of adding neural management to advice to stay active versus only advice to stay active in improving pain intensity, functional limitation, and sciatic neurodynamic of patients with chronic sciatica.

Secondarily, the study aims to assess the relationship between the characteristics of the participants with the self-reported pain intensity. Ultimately, we will analyse whether the effect of the treatment on pain intensity and functional limitation is mediated by sociodemographic factors, pain characteristics, and psychosocial aspects.

Risk and Benefit Assessment:
The risks and benefits of the project are described and well-founded, including actions to be performed in case of adverse events, as follows:

Expected risks
Participants will answer the questionnaires and perform the proposed evaluations. The application of questionnaires present minimal risks related to the embarrassment of not knowing how to answer any item. Participants will be able to complete the questionnaire in the time they deem necessary and may leave items unanswered if they are unable to respond. The responses of participants will be kept confidential. The assessment of Conditioned Pain Modulation (CPT) may cause mild or moderate transiently pain and will be performed in a maximum of 1 minute. If there is any discomfort during the procedures, the research examiners will wait the necessary period with the patient for the patient to return to his/her pre-test sensation. The study researchers will provide the appropriate treatment or referral if the discomfort does not disappear. The use of neural management as a treatment for clinical variables has an immediate effect and no evidence of detrimental effects to the participant (Nee et al., 2012). Thus, the present study presents minimal risk to study participants and the research team.

Expected benefits
This project has considerable relevance due to the high social impact generated by the offer of free service and subsequent follow-up to the population of the metropolitan area of Rio de Janeiro who suffers from chronic sciatica. In addition, both groups are expected to present clinical improvements in your condition, as treatment considered the gold standard will be offered (advice to stay active) for patients with sciatica. It is also expected to contribute to the evidence that makes available the use of neural management plus advice to stay active in clinical practice and future research.

Continuation of Opinion: 4.457.927

Research Comments and Considerations:
The project is very relevant and is very well-founded from an ethical point of view. All the legal documents were presented, and the project presents the risks and benefits of the interventions. In addition, the ICF [Informed Consent Form] is very well contextualised and clear, giving to research participants clarity of all procedures in which they will be submitted.
Considerations for Mandatory Submission Terms:
All mandatory documents were presented:
Project with schedule and budget;
Procedures to be performed;
Risks and Benefits;
Assessment instruments used;
Well-founded ICF [Informed Consent Form] and with clear information for research participants
Recommendations:
This rapporteur opines for the approval of the project
Conclusions or Pending Issues and List of Inadequacies:
CONSIDERING THE CNS RESOLUTION 466/2012;
CONSIDERING THE No. 001/2013;
CONSIDERING THE RDC ANSIVA 39/08;

This rapporteur opines for the approval of the project;
Final Considerations at the discretion of the REC:
The project is approved. It should be noted that the researcher undertakes to attach to Plataforma Brasil a report at the end of the research. Please use the final report template
which can be found on the REC-UNISUAM website (https://www.unisuam.edu.br/pesquisa-extensao-e-inova/pesquisa-e-inovacao/). In addition, in case of an adverse event, it is up to the researcher to report, also through the Plataforma Brasil.

This opinion was prepared based on the documents listed below:

Document Type	File	Post	Author	Situation
Basic information from the project	PB_BASIC_INFORMATIONS_DO_PROJETO_1655066.pdf	18/11/2020 18:25:07		Accepted
Others	Instrumentos.docx	18/11/2020	LETICIA AMARAL	Accepted

[Logo of UNISUAM].
CENTRO UNIVERSITÁRIO AUGUSTO MOTTA [Augusto Motta University Centre].
[Logo of Plataforma Brasil].

Continuation of Opinion: 4.457.927

Others	Instrumentos.docx	18:24:34	CORREA	Accepted
Budget	Orcamento.docx	18/11/2020 18:20:20	LETICIA AMARAL CORREA	Accepted
Schedule	Cronograma.docx	18/11/2020 18:19:21	LETICIA AMARAL CORREA	Accepted
ICF / Assent Terms / Absence Justification	TCLE.docx	18/11/2020 16:49:51	LETICIA AMARAL CORREA	Accepted
Detailed project / Investigator Brochure	Projeto_Doutorado_CEP.docx	18/11/2020 16:49:38	LETICIA AMARAL CORREA	Accepted
Cover Sheet	FolhaDeRosto_LAC.pdf	17/11/2020 19:13:53	LETICIA AMARAL CORREA	Accepted

Status of Opinion:
Approved

Needs Assessment from CONEP [National Research Ethics Commission]:
No

RIO DE JANEIRO, 11 December, 2020

Signed by:
Arthur de Sá Ferreira
(Coordinator)

Address: Rua Dona Isabel, 94, TELEPHONE: (21)3882-9797 (Extension: 9943)
Neighborhood: Bonsucesso **ZIP CODE:** 21.032-060
State: RJ **City:** RIO DE JANEIRO
Telephone: (21)3882-9797 **E-mail:** comitedeetica@souunisuam.com.br
