# Supplementary material for: Neural management plus advice to stay active on clinical measures and sciatic neurodynamic for patients with chronic sciatica: Study protocol for a controlled randomised clinical trial
Source: PLoS One. 2022 Feb 4;17(2):e0263152. doi: 10.1371/journal.pone.0263152 (PMC8815873; doi:10.1371/journal.pone.0263152)
Supplement: S4 File — (PDF) [file pone.0263152.s004.pdf]

PARECER CONSUBSTANCIADO DO CEP

DADOS DO PROJETO DE PESQUISA

Título da Pesquisa: Gerenciamento do tecido neural adicionado a orientação para manter-se ativo em pacientes com ciatalgia crônica.

Pesquisador: LETICIA AMARAL CORREA

Área Temática:

Versão: 1

CAAE: 40500720.8.0000.5235

Instituição Proponente: SOCIEDADE UNIFICADA DE ENSINO AUGUSTO MOTTA

Patrocinador Principal: Financiamento Próprio

DADOS DO PARECER

Número do Parecer: 4.457.927

Apresentação do Projeto:

O projeto de doutorado intitulado "Gerenciamento do tecido neural adicionado a orientação para manter-se ativo nas medidas clínicas e na neurodinâmica do nervo ciático em pacientes com ciatalgia crônica: um ensaio clínico randomizado controlado" fundamenta-se teoricamente que a dor lombar é a principal causa de anos vividos com incapacidade em todo o mundo. Pacientes com ciatalgia apresentam um pior prognóstico quando comparados àqueles com dor lombar localizada. A principal estratégia de tratamento disponível na literatura para esses pacientes é a orientação para manter-se ativo. Outros tratamentos conservadores, como o gerenciamento do tecido neural pode contribuir para uma significativa recuperação da ciatalgia. Contudo, os efeitos do gerenciamento do tecido neural em pacientes com ciatalgia ainda não foram avaliados de forma robusta na literatura. Desta forma os objetivos principal desse projeto é a comparação dos efeitos da adição de gerenciamento do tecido neural a orientação para manter-se ativo versus somente orientação para manter-se ativo na melhora da intensidade de dor, limitação funcional e na neurodinâmica do nervo ciático de pacientes com ciatalgia crônica.

Objetivo da Pesquisa:

Comparar os efeitos da adição de gerenciamento do tecido neural a orientação para manter-se ativo versus somente orientação para manter-se ativo na melhora da intensidade de dor, limitação funcional e na neurodinâmica do nervo ciático de pacientes com ciatalgia crônica.

Endereço: Rua Dona Isabel, 94, TEL: (21)3882-9797 (Ramal: 9943)
Bairro: Bonsucesso **CEP:** 21.032-060
UF: RJ **Município:** RIO DE JANEIRO
Telefone: (21)3882-9797 **E-mail:** comitedeetica@souunisuam.com.br

Secundariamente, o estudo se propõe a avaliar a relação entre as características dos participantes com a intensidade da dor autorreferida. Ultimamente, analisaremos se o efeito do tratamento na intensidade de dor e limitação funcional é mediado por fatores sociodemográficos, características da dor e aspectos psicossociais.

Avaliação dos Riscos e Benefícios:

Os riscos e benefícios do projeto são descritos e bem fundamentados, inclusive com ações a serem executadas em caso de eventos adversos, conforme segue abaixo:

Riscos esperados

Os participantes responderão aos questionários e realizarão as avaliações propostas. A aplicação dos questionários apresenta riscos mínimos relacionados ao constrangimento de não saber responder algum item. Os participantes poderão preencher o questionário no intervalo de tempo que julgar necessário e poderão deixar itens sem resposta, caso não saibam responder. As respostas dos participantes serão mantidas em sigilo. A avaliação da modulação condicionada da dor (CPT) pode causar dor leve ou moderada de forma transitória e será realizada em no máximo 1 minuto. Caso haja algum desconforto durante os procedimentos, os examinadores da pesquisa aguardarão o período necessário junto ao paciente para que o paciente retorne à sua sensação pré-teste. Os pesquisadores do estudo irão fornecer o adequado tratamento ou encaminhamento caso os desconfortos não desapareçam. O uso do gerenciamento do tecido neural como tratamento para variáveis clínicas apresenta efeito imediato e sem evidências de efeitos prejudiciais ao participante (Nee et al., 2012). Dessa forma, o presente estudo apresenta mínimos riscos para os participantes do estudo e para a equipe de pesquisa.

Benefícios esperados

O presente projeto possui considerável relevância graças ao elevado impacto social gerado pela oferta de atendimento gratuito e subsequente acompanhamento à população da zona metropolitana do Rio de Janeiro que sofre com ciatalgia crônica. Além disso, espera-se que ambos os grupos apresentem melhoras clínicas em sua condição, visto que será ofertado tratamento considerado padrão-ouro (orientação a manter-se ativo) para pacientes com ciatalgia. Espera-se também contribuir para as evidências que viabilizam o uso de gerenciamento do tecido neural adicionado a orientação para manter-se ativo na prática clínica e em futuras pesquisas.

Endereço: Rua Dona Isabel, 94, TEL: (21)3882-9797 (Ramal: 9943)
Bairro: Bonsucesso **CEP:** 21.032-060
UF: RJ **Município:** RIO DE JANEIRO
Telefone: (21)3882-9797 **E-mail:** comitedeetica@souunisuam.com.br

Comentários e Considerações sobre a Pesquisa:

O projeto é bastante relevante e está muito bem fundamentado do ponto de vista ético. Todos os documentos legais foram apresentados e o projeto apresenta os riscos e benefícios das intervenções. Além disso, o TCLE está muito bem contextualizado e claro, deixando aos participantes da pesquisa clareza de todos os procedimentos em que eles serão submetidos.

Considerações sobre os Termos de apresentação obrigatória:

Todos os documentos obrigatórios foram apresentados:
Projeto com cronograma e orçamento;
Procedimentos a serem executados;
Riscos e Benefícios;
Instrumentos avaliativos utilizados;
TCLE bem fundamentado e com clareza de informações para os participantes da pesquisa

Recomendações:

Este relator opina pela aprovação do projeto

Conclusões ou Pendências e Lista de Inadequações:

CONSIDERANDO A RESOLUÇÃO CNS 466/2012;
CONSIDERANDO A no 001/2013;
CONSIDERANDO A RDC ANSIVA 39/08;

Este relator opina pela aprovação do projeto;

Considerações Finais a critério do CEP:

O projeto está aprovado. Cabe ressaltar que o pesquisador se compromete em anexar na Plataforma Brasil um relatório ao final da realização da pesquisa. Pedimos a gentileza de utilizar o modelo de relatório final que se encontra na página eletrônica do CEP-UNISUAM (https://www.unisuam.edu.br/pesquisa-extensao-e-inova/pesquisa-e-inovacao/). Além disso, em caso de evento adverso, cabe ao pesquisador relatar, também através da Plataforma Brasil.

Este parecer foi elaborado baseado nos documentos abaixo relacionados:

Tipo Documento	Arquivo	Postagem	Autor	Situação
Informações Básicas do Projeto	PB_INFORMAÇÕES_BÁSICAS_DO_PROJETO_1655066.pdf	18/11/2020 18:25:07		Aceito
Outros	Instrumentos.docx	18/11/2020	LETICIA AMARAL	Aceito

Outros	Instrumentos.docx	18:24:34	CORREA	Aceito
Orçamento	Orcamento.docx	18/11/2020 18:20:20	LETICIA AMARAL CORREA	Aceito
Cronograma	Cronograma.docx	18/11/2020 18:19:21	LETICIA AMARAL CORREA	Aceito
TCLE / Termos de Assentimento / Justificativa de Ausência	TCLE.docx	18/11/2020 16:49:51	LETICIA AMARAL CORREA	Aceito
Projeto Detalhado / Brochura Investigador	Projeto_Doutorado_CEP.docx	18/11/2020 16:49:38	LETICIA AMARAL CORREA	Aceito
Folha de Rosto	FolhaDeRosto_LAC.pdf	17/11/2020 19:13:53	LETICIA AMARAL CORREA	Aceito

Situação do Parecer:
Aprovado

Necessita Apreciação da CONEP:
Não

RIO DE JANEIRO, 11 de Dezembro de 2020

Assinado por:
Arthur de Sá Ferreira
(Coordenador(a))
